# Adult‐Onset Compared to Childhood‐Onset Asthma: Multifaceted Symptoms, Comorbidity, and Healthcare Burden

**DOI:** 10.1002/clt2.70160

**Published:** 2026-02-14

**Authors:** Reshed Abohalaka, Selin Ercan, Lauri Lehtimäki, Daniil Lisik, Saliha Selin Ozuygur Ermis, Helena Backman, Madeleine Rådinger, Bright I. Nwaru, Hannu Kankaanranta

**Affiliations:** ^1^ Department of Internal Medicine and Clinical Nutrition Krefting Research Centre Institute of Medicine Sahlgrenska Academy University of Gothenburg Gothenburg Sweden; ^2^ Allergy Centre Tampere University Hospital Tampere Finland; ^3^ Faculty of Medicine and Health Technology Tampere University Tampere Finland; ^4^ Department of Public Health and Clinical Medicine Umeå University Umeå Sweden; ^5^ Department of Respiratory Medicine Seinäjoki Central Hospital Seinäjoki Finland

## Abstract

In this study, we compared symptoms, comorbidities, and healthcare burden between childhood‐onset asthma (< 18 years) and early adult‐onset (18–39 years) and late adult‐onset asthma (≥ 40 years). Among 3546 participants with data on physician‐diagnosed asthma and onset age, 46.4% were defined as adult‐onset [864 (24.4%) had early adult‐onset asthma (18–39 years) and 782 (22.1%) had late adult‐onset asthma (≥ 40 years)], which, compared to childhood‐onset asthma, presented with more complex, multi‐symptom profiles, including productive cough, sputum production, but less wheezing. Allergy‐related comorbidities were more common in childhood‐diagnosed asthma, while chronic obstructive pulmonary disease (COPD), diabetes mellitus, hypertension, obesity, and chronic sinusitis were more common in adult‐onset asthma. Adult‐onset asthma also had a higher disease burden, with more frequent medication use and exacerbations. Adult‐onset asthma has an underlying complexity, contributing to a vicious cycle of worsening symptoms, increased medication use, and more comorbidities.

To the editor,

Asthma varies by age and has often more severe outcomes when its onset begins in adulthood than childhood [1, 2, 3]. Limited population‐based studies suggest that adult‐diagnosed asthma presents with different symptoms and comorbidities than childhood‐diagnosed [4, 5]. However, most studies overlook delayed diagnosis and exclude older adults, potentially misrepresenting the nature of adult‐onset asthma. Large, representative studies focusing on age at onset are needed to advance our understanding of differences between childhood‐ and adult‐onset asthma.

We used survey data from the West Sweden Asthma Study (WSAS) [6]. In 2008 and 2016, WSAS invited 80,000 randomly selected adults from the general population to complete a postal questionnaire on asthma, respiratory symptoms, allergies, and various environmental exposures. A total of 42,621 responded, and 3546 reported having physician‐diagnosed asthma along with their onset age (Supporting Information S1: Figure S1). We calculated the percentage of those experiencing: nine symptoms (*dyspnea, dyspnea with wheezing, any wheezing, wheezing without having cold, long‐lasting cough, productive cough, sputum production, rhinorrhea, and waking up due to cough, chest tightness or dyspnea*), seven comorbidities (*chronic obstructive pulmonary disease (COPD), obesity, allergic rhinitis, chronic sinusitis, hypertension, diabetes mellitus, and eczema*), and four measures of asthma healthcare burden (*ever using asthma medication, medication use past 12 months, asthma exacerbations past 12 months, and ever hospitalization due to asthma*), separately and cumulatively for childhood‐onset (defined as onset < 18 years), early adult‐onset (defined as onset 18–39 years), and late adult‐onset (defined as onset ≥ 40 years) asthma participants. In addition, we fitted multivariable modified Poisson regression models to estimate adjusted risk ratios (RRs) for the association between age at asthma onset (childhood‐ vs. early adult‐onset and late adult‐onset asthma) and each outcome, adjusting for age, gender, education, and smoking.

Of the 3546 asthma participants, 1646 (46.4%) had adult‐onset asthma [864 (24.4%) had early adult‐onset asthma (18–39 years) and 782 (22.1%) had late adult‐onset asthma (≥ 40 years)]. Both early adult‐onset and late adult‐onset asthma participants were older, less often current smokers but more often ever smokers with higher total pack‐years, had a higher BMI, and included more women than those with childhood‐onset asthma (Table 1). Allergy‐related comorbidities, such as allergic rhinitis and eczema, were more common in childhood‐onset asthma. In contrast, COPD, diabetes, hypertension, obesity, chronic sinusitis, and having > 2 comorbidities were more common in early adult‐onset and late adult‐onset asthma (Figure 1).

**TABLE 1:** Characteristics among participants with childhood‐onset and adult‐onset asthma.

	All asthma participants (*n* = 3546)				Men (*n* = 1413)				Women (*n* = 2133)			
	Childhood‐onset asthma (< 18 years) (*n* = 1900)	Adult‐onset asthma (≥ 18 years) (*n* = 1646)		*p* value	Childhood‐onset asthma (< 18 years) (*n* = 848)	Adult‐onset asthma (≥ 18 years) (*n* = 565)		*p* value	Childhood‐onset asthma (< 18 years) (*n* = 1052)	Adult‐onset asthma (≥ 18 years) (*n* = 1081)		*p* value
		Early adult‐onset (18–39) years (*n* = 864)	Late adult‐onset (≥ 40 years) (*n* = 782)			Early adult‐onset (18–39) years (*n* = 290)	Late adult‐onset (≥ 40 years) (*n* = 275)			Early adult‐onset (18–39) years (*n* = 574)	Late adult‐onset (≥ 40 years) (*n* = 507)	
Gender (men)	848 (44.6%)	290 (33.6%)	275 (35.2%)	**<** **0.001**								
Age (y)	35 (16–76)	46 (19–76)	64 (41–76)	**<** **0.001**	36 (16–76)	47 (22–76)	64 (41–76)	**<** **0.001**	34 (16–76)	45 (19–76)	64 (41–76)	**<** **0.001**
Age at asthma onset (y)	8 (0–17)	26 (18–39)	50 (40–76)	**<** **0.001**	7 (0–17)	25 (18–39)	50 (40–76)	**<** **0.001**	10 (0–17)	27 (18–39)	50 (40–75)	**<** **0.001**
BMI (kg/m^2^)	24.4 (9–53)	25.5 (16–51)	27.1 (16.6–56)	**<** **0.001**	25.1 (9–43)	26.3 (16.3–45)	26.9 (19.6–56)	**<** **0.001**	23.8 (12.5–53)	25 (16–51)	27.2 (16.6–50)	**<** **0.001**
Education (≥ 12 y)	824 (43.8%)	369 (43.4%)	264 (34.6%)	**<** **0.001**	327 (38.9%)	94 (32.6%)	85 (31.8%)	**0.039**	497 (47.7%)	275 (48.8%)	179 (36.1%)	**<** **0.001**
Current smokers	281 (14.9%)	110 (13.1%)	83 (10.8%)	**0.002**	119 (14.1%)	25 (8.8%)	26 (9.6%)	**0.100**	162 (15.6%)	85 (15.3%)	57 (11.5%)	**0.006**
Ever smokers	377 (20.9%)	223 (27.4%)	326 (43.1%)	**<** **0.001**	168 (20.6%)	96 (34.3%)	125 (46.1%)	**<** **0.001**	209 (21.1%)	127 (23.8%)	201 (41.4%)	**<** **0.001**
Pack year	7.6 (0–100)	10 (0–107.8)	18 (0–110)	**0.004**	9.5 (0–76)	11.2 (0–107.8)	21 (0–110)	0.191	7 (0–100)	9.5 (0–84)	16.7 (0.2–80)	0.085

*Note:* Data is presented as *n* (%) or median (min‐max). Bold font indicates *p* < 0.05 which was considered statistically significant.

Abbreviation: WSAS, West Sweden Asthma Study.

**FIGURE 1:**
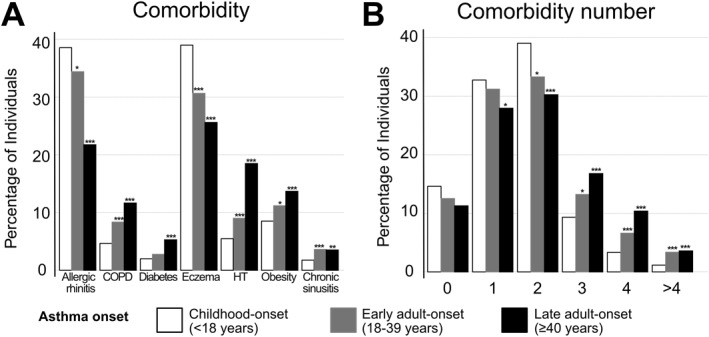
Percentage of individuals with selected asthma comorbidities (COPD, obesity, allergic rhinitis, chronic sinusitis, hypertension, diabetes mellitus, and eczema) categorized by age at asthma onset (< 18, 18–39, and ≥ 40). (A) Denotes the prevalence of individual measures, while (B) denote the number of such measures present. Proportion tests (chi‐square tests for equality of proportions with continuity correction) were used where assumptions were met; otherwise, Fisher's exact tests were applied. Raw *p*‐values were adjusted using the Holm method. COPD, chronic obstructive pulmonary disease; HT, hypertension. * = *p* < 0.05, ** = *p* < 0.01, *** = *p* < 0.001.

Late adult‐onset asthma participants had a higher prevalence of sputum production, and both early adult‐onset and late adult‐onset asthma participants had a higher prevalence of productive cough than those with childhood‐onset asthma. Having no symptoms was more common in childhood‐onset asthma, while having ≥ 5 symptoms was more common in early adult‐onset and late adult‐onset asthma (Figure 2A,B). Individuals with childhood‐onset asthma had more lifetime hospitalizations due to asthma and fewer than one measure of asthma healthcare burden. In contrast, those with early adult‐onset and late adult‐onset asthma had higher medication use, and those with early adult‐onset had more exacerbations in the past 12 months. Adult‐onset asthma participants had high proportion with ≥ 2 measures of asthma healthcare burden (Figure 2C,D). The results of the regression analysis showed that compared to childhood‐onset, late adult‐onset asthma had a higher risk of having all symptoms, all types of healthcare burden (except hospitalization), comorbidities such as chronic sinusitis and obesity. Both early adult‐onset and late adult‐onset asthma were associated with having two or more symptoms or healthcare burden (Figure 3). For analysis regarding early adult‐onset and late adult‐onset asthma combined versus childhood‐onset asthma, see Supporting Information S1: Figures S2–S4.

**FIGURE 2:**
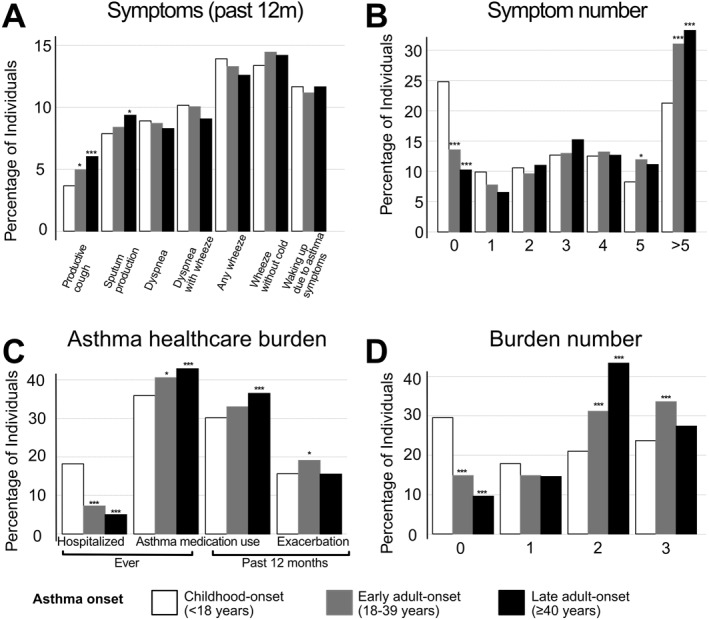
Percentage of individuals with selected asthma symptoms (dyspnea, dyspnea with wheezing, any wheezing, wheezing without having cold, productive cough, sputum production, and waking up due to cough, chest tightness or dyspnea) and measures of healthcare burden (ever using asthma medication, medication use past 12 months, asthma exacerbations past 12 months, and ever hospitalization due to asthma), categorized by age at asthma onset (< 18, 18–39, and ≥ 40). (A) and (C) denotes the prevalence of individual measures for asthma symptoms and healthcare burden, respectively. (B) and (D) denote the number of such measures present, respectively. Proportion tests (chi‐square tests for equality of proportions with continuity correction) were used where assumptions were met; otherwise, Fisher's exact tests were applied. Raw *p*‐values were adjusted using the Holm method. Waking up due to asthma symptoms = waking up due to cough, chest tightness or dyspnea, * = *p* < 0.05, ** = *p* < 0.01, *** = *p* < 0.001.

**FIGURE 3:**
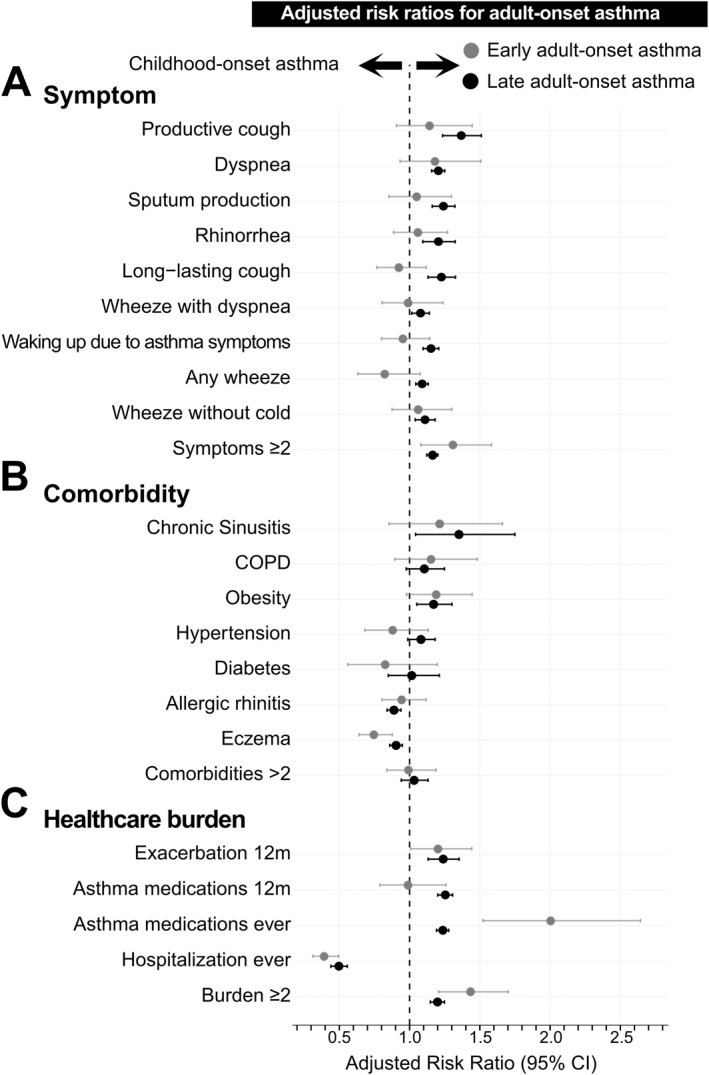
Multivariable modified Poisson regression models with robust standard errors to estimate adjusted risk ratios (RRs) and 95% confidence intervals (CIs) for each outcome. The main exposure was age at asthma onset (Early adult‐onset asthma (18–39 years) versus childhood‐onset asthma & Late adult‐onset asthma (≥ 40 years) versus childhood‐onset asthma), using childhood‐onset asthma as the reference group. All estimates were adjusted for age, gender, education, and smoking status. Outcomes are (A): symptoms, (B) comorbidities, and (C) healthcare burden indicators. Waking up due to asthma symptoms = waking up due to cough, chest tightness or dyspnea.

In summary, our results show that some individual symptoms, comorbidities, and measures of healthcare burden may be less common in early adult‐onset or late adult‐onset than childhood‐onset asthma. However, adult‐onset asthma has a more complex picture with multiple symptoms, comorbidity and healthcare burden. Though mislabeling the symptoms of other disorders can't be ruled out due to our study design, higher medication use suggests true disease and healthcare burden. Therefore, our study suggests a vicious cycle where adult‐onset asthma may share molecular mechanisms with other chronic diseases, worsening symptoms and increasing the need for medication, which could, in turn, contribute to the development of more chronic disorders, even after adjusting for age and other covariates. While age undoubtedly contributes to the higher prevalence of these conditions, our findings suggest that intrinsic asthma is linked to an excess burden of metabolic and cardiovascular comorbidities that cannot be explained by age alone. This pattern resembles what was described many years ago as intrinsic asthma [7, 8, 9].

## Author Contributions

**Conception and design:** Reshed Abohalaka, Lauri Lehtimäki, Bright I. Nwaru, Hannu Kankaanranta. **Data analysis:** Reshed Abohalaka, Selin Ercan, Helena Backman, Bright I. Nwaru, Hannu Kankaanranta. **Data collection:** Reshed Abohalaka, Selin Ercan, Daniil Lisik, Saliha Selin Ozuygur Ermis, Madeleine Rådinger, Bright I. Nwaru, Hannu Kankaanranta. **Manuscript writing:** Reshed Abohalaka, Hannu Kankaanranta. Manuscript review and editing all authors.

## Conflicts of Interest

RA reports travel grants from the American Thoracic Society (ATS), the European Respiratory Society (ERS), the Swedish Heart and Lung Foundation (HLF), and the Adlerbertska foundations, outside the current work. HK reports fees for lectures and/or consulting from AstraZeneca, Boehringer‐Ingelheim, Chiesi Pharma, Covis Pharma, GSK, MSD, Orion Pharma and Sanofi outside the current study. The rest of the authors have no conflict of interest to declare.

## Supporting information


Supporting Information S1

## Data Availability

Data is classified sensitive and protected and processed according to GDPR rules of European Union.
