# Supplementary material for: Adult‐Onset Compared to Childhood‐Onset Asthma: Multifaceted Symptoms, Comorbidity, and Healthcare Burden
Source: Clin Transl Allergy. 2026 Feb 14;16(2):e70160. doi: 10.1002/clt2.70160 (PMC12906355; doi:10.1002/clt2.70160)
Supplement: Supplementary file 1 — Supporting Information S1 [file CLT2-16-e70160-s001.docx]

Adult-onset Compared to Childhood-onset Asthma: Multifaceted Symptoms, Comorbidity, and Healthcare Burden

Reshed Abohalaka^a^, Selin Ercan^a^, Lauri Lehtimäki^b,c^, Daniil Lisik^a^, Saliha Selin Ozuygur Ermis^a^, Helena Backman^d^, Madeleine Rådinger^a^, Bright I. Nwaru^a^, Hannu Kankaanranta^a,c,e^

^a^Krefting Research Centre, Department of Internal Medicine and Clinical Nutrition, Institute of Medicine, Sahlgrenska Academy, University of Gothenburg, Gothenburg, Sweden

^b^Allergy Centre, Tampere University Hospital, Tampere, Finland

^c^Faculty of Medicine and Health Technology, Tampere University, Tampere, Finland

^d^Department of Public Health and Clinical Medicine, Umeå University, Umeå, Sweden

^e^Department of Respiratory Medicine, Seinäjoki Central Hospital, Seinäjoki, Finland

Corresponding author: Reshed Abohalaka, MSc

Krefting Research Centre

Department of Internal Medicine and Clinical Nutrition

Institute of Medicine

Sahlgrenska Academy

Medicinaregatan 1F, Box 424, University of Gothenburg, 405 30 Gothenburg, Sweden

Tel: +46 31 786 67 12

e-mail: reshed.abohalaka@gu.se

**Supplementary file
Methods**

***Study population***

The West Sweden Asthma Study (WSAS) has been described in detail in a previous publication.^1^ To summarize, WSAS encompasses an initial cross-sectional examination of individuals aged 16 to 75 years. The inaugural survey was conducted in 2008, wherein a cohort of 30,000 individuals, selected at random to mirror the age and gender distribution of the study region, were solicited for participation. Of the initially targeted survey respondents, excluding those that were untraceable (n=782), 18,087 individuals participated in the survey. A second survey was executed in 2016, extending invitations to a randomly selected group of 50,000 individuals within the same age range as those included in the 2008 survey, with no overlapping participants between the two surveys. Overall, 24,534 individuals among the 50,000 invitees participated in the 2016 survey. Combining the participants from the two surveys, the total number of participants was 42,621 **(Figure S1**). All participants provided an informed consent. Approval for the study was granted by the Regional Ethics Committee of Gothenburg, Sweden.

***Data collection and questionnaire***

At the outset of the study, participants were administered a postal self-administered questionnaire, which consisted of questions previously employed in various epidemiological investigations including the Obstructive Lung Diseases in Northern Sweden (OLIN),^2,3^ the Global Allergy and Asthma European Network (GA^2^LEN) studies, the FinEsS studies conducted in Finland, Estonia, and Sweden,^4,5^ and the European Community Respiratory Health Survey (ECRHS).^6^ Questions derived from the OLIN and ECRHS surveys primarily addressed topics related to asthma, rhinitis, chronic bronchitis, chronic obstructive pulmonary disease (COPD), emphysema, respiratory symptoms, asthma medication usage, and potential risk factors encompassing smoking habits, family history of respiratory illnesses, occupational type, occupational and environmental exposures, comorbidities, and socioeconomic status. Furthermore, the GA^2^LEN questionnaire was integrated to incorporate additional detailed inquiries regarding rhinosinusitis and eczema. Prior to implementation in the study, the questionnaire was translated into Swedish.

***Definitions of morbidity***

We have analysed total of seven comorbidities (*chronic obstructive pulmonary disease (COPD), obesity, allergic rhinitis, chronic sinusitis, hypertension, diabetes mellitus, and eczema*) in asthma participants. *Asthma* was defined as a positive response to the question*, "Have you been diagnosed by a physician as having asthma?"* Subsequently, the age at which asthma was started was determined through a follow-up question, *"How old were you when you got asthma? "*. Individuals reporting asthma onset beyond their current age, or those affirming physician-diagnosed asthma but failing to provide the age of onset (*n*=429), were excluded from the analysis. *Childhood-onset asthma* was defined as onset <18 years, *early adult-onset asthma* as onset between 18-39 years, and *late adult-onset asthma* as onset ≥40 years.

Comorbidities were defined as the following: **COPD** was defined as a positive response to the question *"Have you been diagnosed by a physician as having COPD or emphysema?".* **Obesity** was defined as having BMI ≥ 30 kg/m^2^. **Allergic rhinitis** was defined as a positive response to the question *"Have you ever had allergic rhinitis?".* **Chronic sinusitis** was defined as a positive response to the question *"Have you been diagnosed by a physician as having chronic sinusitis?".* **Hypertension** *and* **diabetes mellitus** were defined a positive response to the question*, "Do you currently use medication for hypertension?"* and *"Do you currently use medication for diabetes?",* respectively*.* ***Eczema*** was defined a positive response to the question *"Have you ever had skin rashes or eczema?".*

***Definitions of measures of asthma healthcare burden***

We analysed four measures of asthma healthcare burden defined as follows: **Use of asthma medication in the past year** was evaluated by a “yes” to the question *“Do you currently or in the past year use asthma medications?”.* **Use of asthma medications ever** was evaluated by a “yes” to the question *“Have you ever used asthma medications?”*. **Asthma exacerbations in the past year** were defined as a “yes” to the question *“Have you had an asthma attack in the past 12 months?”*. **Lifetime hospitalisation** was defined as a “yes” to the question *“Have you ever been hospitalised due to asthma?”*.

***Definitions of asthma symptoms***

The following nine asthma-related symptoms were assessed: **dyspnea**, **any** **wheezing**, **wheezing with dyspnea**, **wheezing without having cold**, **long-lasting cough,** **productive cough**, **sputum production**, **long-lasting rhinorrhea**, and **waking up due to cough, chest tightness or dyspnea**. The symptoms were assessed based on a “yes” to the following questions;

“*Have you, during the last 12 months, had short of breath?*”,

“*Have you, during the last 12 months, had any wheezing?*”,

“*Have you, during the last 12 months, had wheezing with short of breath?*”,

“*Have you, during the last 12 months, had wheezing without having cold?*”,

“*Have you, during the last 12 months, woken up with cough, tight chest or short of breath?*”, “*Have you, during the last 12 months, had nasal obstruction or rhinorrhea for more than 12 weeks?*”,

*“Have you, during the last 12 months, had long-lasting cough?”.*

“*Have you, during the last 12 months, had productive cough lasting more than 3 months?”.*
“*Have you, during the last 12 months, had sputum production?”.*

***Statistical analysis***

Statistical analyses were conducted utilizing R language (R version 4.5.0). Chi-square test was used to compare distribution of categorical variables in participants with early adult-onset and late adult-onset vs childhood-onset asthma. Proportion tests (chi-square tests for equality of proportions with continuity correction) were used where assumptions were met; otherwise, Fisher’s exact tests were applied. To account for multiple pairwise tests, raw *p*-values were adjusted using the Holm method, which controls the familywise error rate. Adjusted p-values were reported. We compared continuous variables across the three study groups defined by age at asthma onset using the Kruskal–Wallis test.

We fitted multivariable modified Poisson regression models with robust standard errors to estimate adjusted risk ratios (RRs) and 95% confidence intervals (CIs) for association between age at asthma onset (childhood- vs. early adult-onset asthma) and (childhood- vs. late adult-onset asthma) and each outcome; childhood-onset asthma was used as the reference exposure group. All models were adjusted for age, gender, education, and smoking status. The modified Poisson regression model approach provides valid estimates of relative risk for binary outcomes and is preferred over logistic regression when the outcome is common ^7^. Results were visualized using forest plots showing adjusted RRs and their 95% CIs by outcome group. *P* values below 0.05 considered statistically significant.


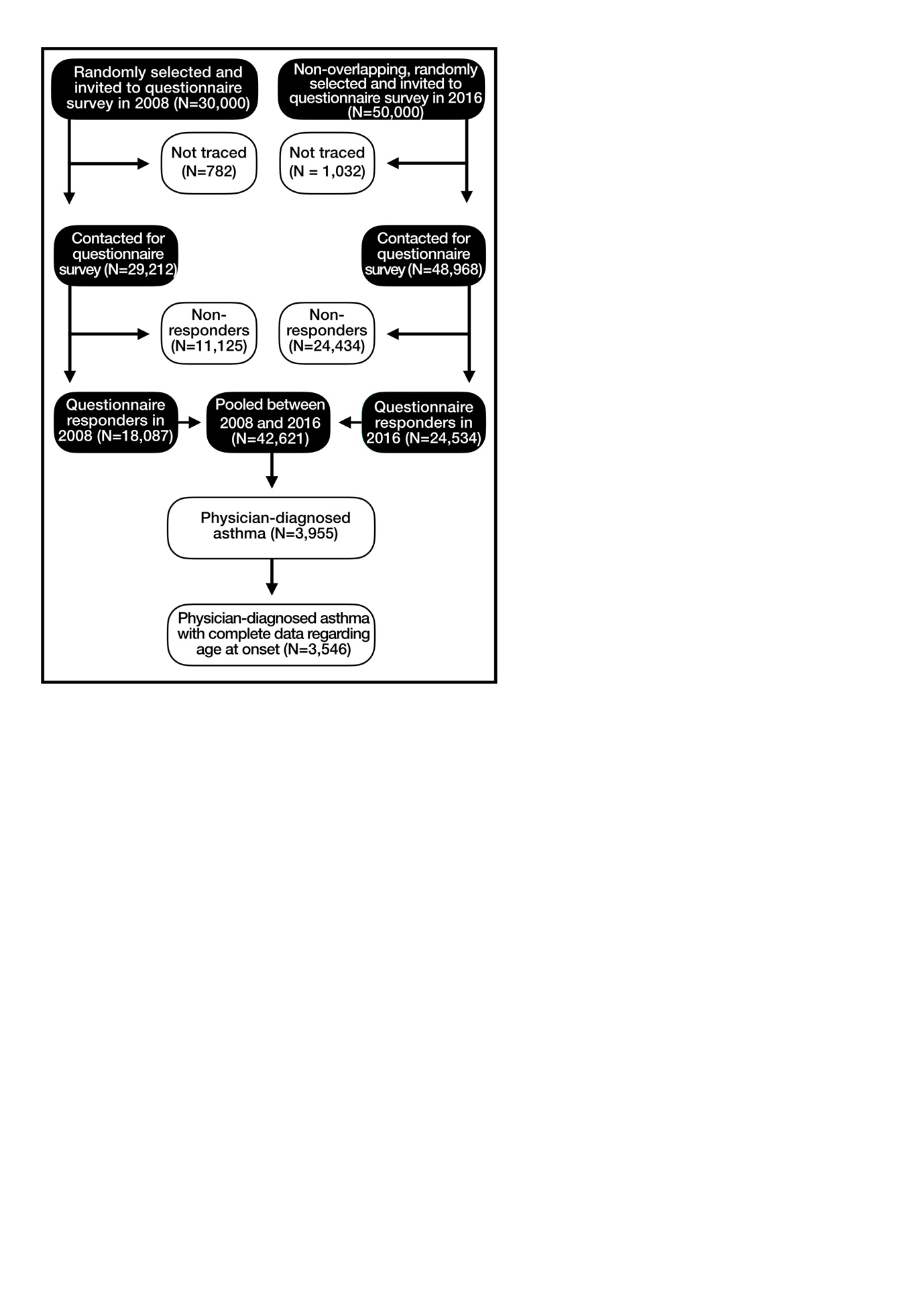


**Figure S1.** Flow chart of the West Sweden Asthma Study (WSAS).

Results:


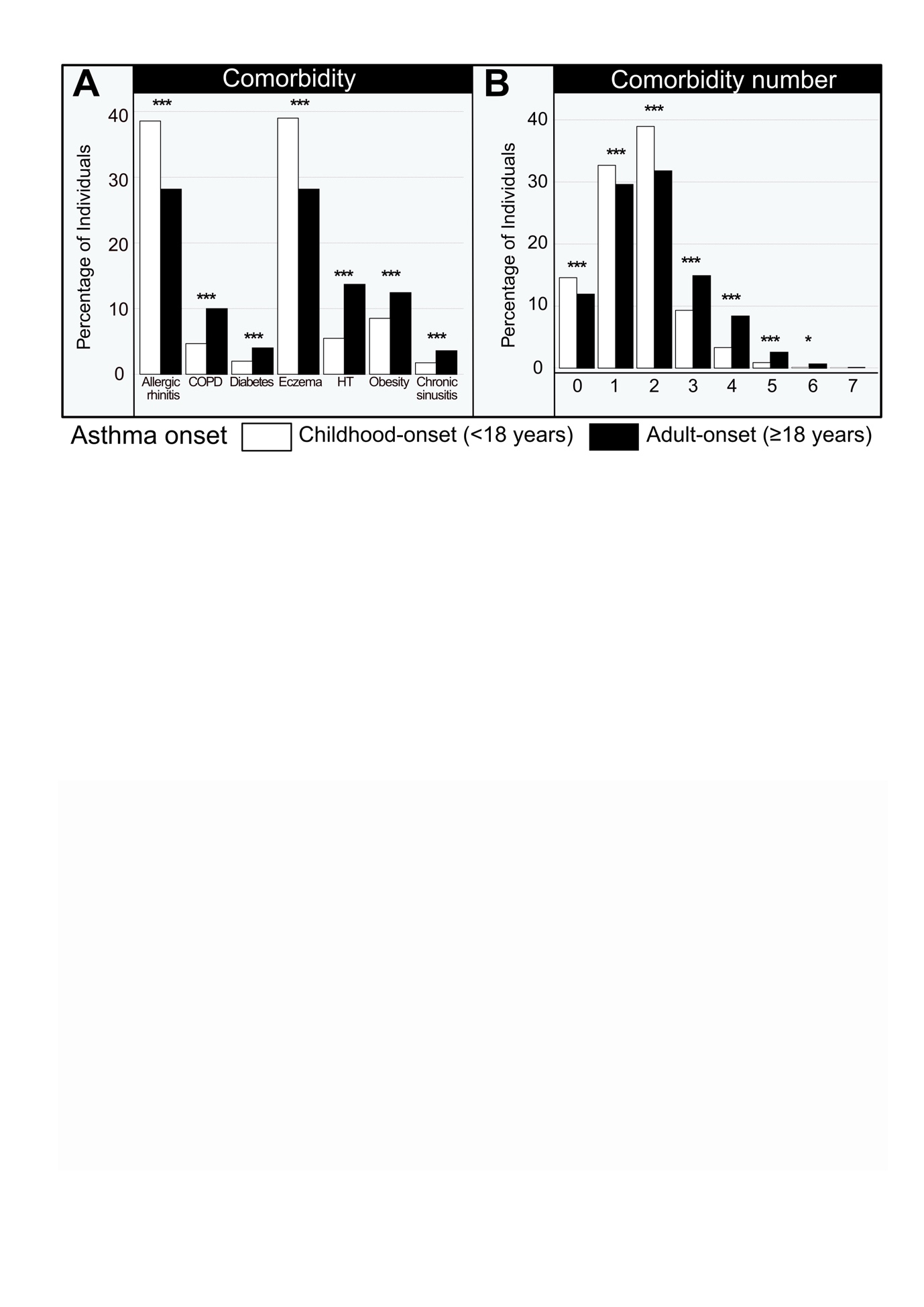


**Figure S2.** Percentage of individuals with selected asthma comorbidities (COPD, obesity, allergic rhinitis, chronic sinusitis, hypertension, diabetes mellitus, and eczema) categorized by age at asthma onset (<18 and ≥18). (A) denotes the prevalence of individual measures, while (B) denote the number of such measures present. Proportion tests (chi-square tests for equality of proportions with continuity correction) were used where assumptions were met; otherwise, Fisher’s exact tests were applied. raw p-values were adjusted using the Holm method. Notes: COPD = chronic obstructive pulmonary disease, HT = hypertension, * = *p*<0.05, ** = *p*<0.01, *** = *p*<0.001.


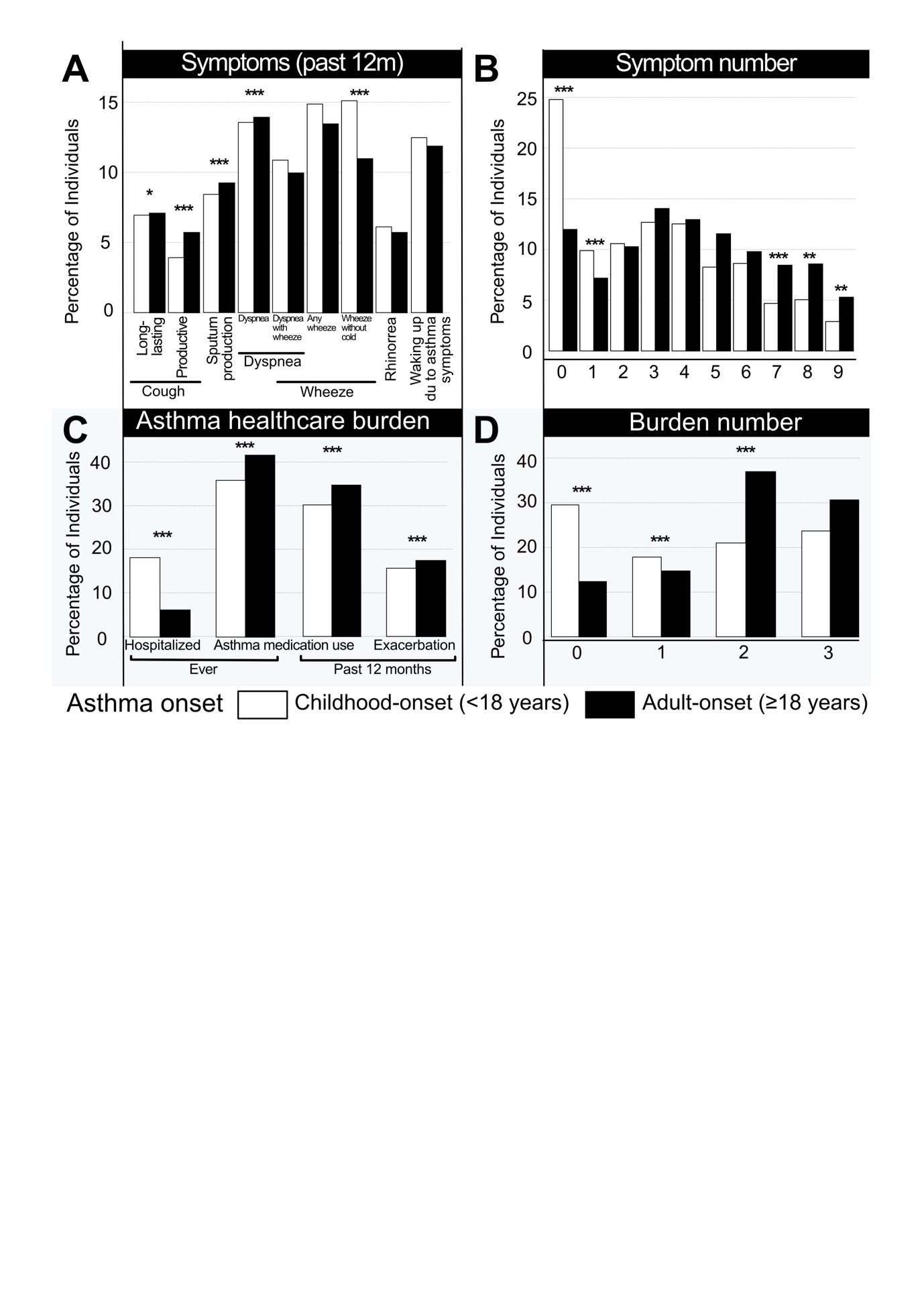


Figure S3: Percentage of individuals with (A) selected asthma symptoms (dyspnea, dyspnea with wheezing, any wheezing, wheezing without having cold, long-lasting cough, productive cough, sputum production, rhinorrhea, and waking up due to cough, chest tightness or dyspnea) and (B) measures of healthcare burden (ever using asthma medication, medication use past 12 months, asthma exacerbations past 12 months, and ever hospitalization due to asthma), categorized by age at asthma onset (<18 and ≥18). (A) and (C) denotes the prevalence of individual measures for asthma symptoms and healthcare burden, respectively. (B) and (C) denote the number of such measures present, respectively. Proportion tests (chi-square tests for equality of proportions with continuity correction) were used where assumptions were met; otherwise, Fisher’s exact tests were applied. raw p-values were adjusted using the Holm method. Notes: Waking up due to asthma symptoms = waking up due to cough, chest tightness or dyspnea, * = *p*<0.05, ** = *p*<0.01, *** = *p*<0.001.


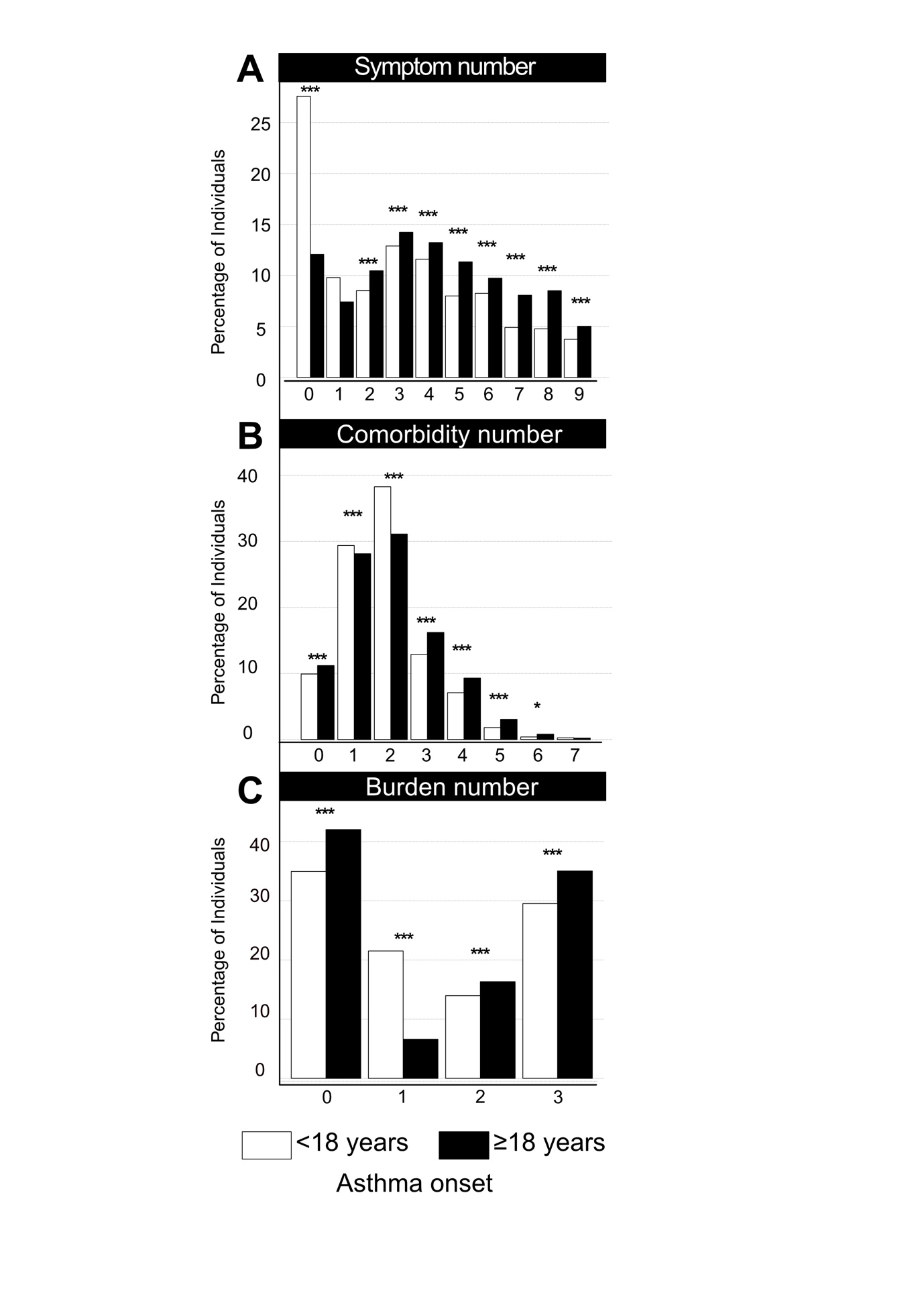


Figure S4: Percentage of individuals who are above 40 years old with (A) number of selected asthma symptoms (dyspnea, dyspnea with wheezing, any wheezing, wheezing without having cold, long-lasting cough, productive cough, sputum production, rhinorrhea, and waking up due to cough, chest tightness or dyspnea), (B) number of selected asthma comorbidities (COPD, obesity, allergic rhinitis, chronic sinusitis, hypertension, diabetes mellitus, and eczema), and (C) number of measures of healthcare burden (ever using asthma medication, medication use past 12 months, asthma exacerbations past 12 months, and ever hospitalization due to asthma), categorized by age at onset before or after the age of 18 years. Chi-square test was used for significance comparison. Notes: Waking up due to asthma symptoms = waking up due to cough, chest tightness or dyspnea, * = *p*<0.05, ** = *p*<0.01, *** = *p*<0.001.

Discussion:

**Study limitations**

Several limitations of our study should be noted. First, the study relies on self-reported data from the WSAS survey, which may introduce recall bias or misclassification of disease onset, comorbidities, and symptoms. For example, some participants classified as having asthma may have had symptoms due to other respiratory or chronic conditions. Although this cannot be fully excluded, the consistently higher medication use in the adult-onset group suggests that the reported burden reflects true disease.

Second, while we used age at onset to classify asthma phenotypes, the cut-off values are not absolute, and some degree of mislabelling between childhood- and adult-onset asthma is possible. Similarly, although COPD was reported by a proportion of participants with childhood-onset asthma, it is important to emphasize that all participants were adults at the time of the survey, making a diagnosis of COPD in this subgroup clinically plausible.

Third, our cross-sectional design limits the ability to determine causality between asthma, comorbidities, and medication burden. We cannot exclude the possibility that some of the associations reflect reverse causation or shared underlying mechanisms. Age, smoking, and socioeconomic factors may also contribute, although we attempted to account for these through adjustment.

Finally, despite the large sample size, the generalizability of our findings may be limited to populations similar to Sweden in terms of healthcare access and environmental exposures. Longitudinal studies with clinical validation of diagnoses and mechanistic biomarkers are needed to strengthen causal inference and to disentangle age-related effects from asthma-specific comorbidity patterns more clearly.

**References:**

1. Nwaru BI, Ekerljung L, Rådinger M, et al. Cohort profile: the West Sweden Asthma Study (WSAS): a multidisciplinary population-based longitudinal study of asthma, allergy and respiratory conditions in adults. *BMJ Open*. Jun 19 2019;9(6):e027808. doi:10.1136/bmjopen-2018-027808

2. Rönmark E, Jönsson E, Lundbäck B. Remission of asthma in the middle aged and elderly: report from the Obstructive Lung Disease in Northern Sweden study. *Thorax*. Jul 1999;54(7):611-3. doi:10.1136/thx.54.7.611

3. Rönmark E, Lundbäck B, Jönsson E, Jonsson AC, Lindström M, Sandström T. Incidence of asthma in adults--report from the Obstructive Lung Disease in Northern Sweden Study. *Allergy*. Nov 1997;52(11):1071-8. doi:10.1111/j.1398-9995.1997.tb00178.x

4. Lindström M, Kotaniemi J, Jönsson E, Lundbäck B. Smoking, respiratory symptoms, and diseases : a comparative study between northern Sweden and northern Finland: report from the FinEsS study. *Chest*. Mar 2001;119(3):852-61. doi:10.1378/chest.119.3.852

5. Pallasaho P, Lindström M, Põlluste J, Loit HM, Sovijärvi A, Lundbäck B. Low socio-economic status is a risk factor for respiratory symptoms: a comparison between Finland, Sweden and Estonia. *Int J Tuberc Lung Dis*. Nov 2004;8(11):1292-300.

6. Burney PG, Luczynska C, Chinn S, Jarvis D. The European Community Respiratory Health Survey. *Eur Respir J*. May 1994;7(5):954-60. doi:10.1183/09031936.94.07050954

7. Zou G. A modified poisson regression approach to prospective studies with binary data. *Am J Epidemiol*. Apr 1 2004;159(7):702-6. doi:10.1093/aje/kwh090
